# Twenty-fourth Annual Report of the Middletown State Homœopathic Hospital at Middletown, N. Y.

**Published:** 1895-11

**Authors:** 


Twenty-Fourth Annual Report of the Middletown State Homœopathic Hospital at Middletown, New York. Transmitted to the Legislature, January, 1895. Albany: James B. Lyon, State Printer. 1895.

This book gives, as its title indicates, a complete account of the administration of the hospital for the preceding year 1894.

For the information of those members of the profession who are not familiar with this institution, it may be stated that it is a hospital for the treatment of the insane by homœopathic methods, supported by the State of New York. For several years the medical superintendent and physician in chief has been the distinguished homœopathic physician, Selden H. Talcott, A. M., M. D., Ph. D. He still holds that position.

In addition to the usual statements concerning the hospital found in all such reports, the present volume contains several interesting medical essays, only the titles of which we can give in the present notice. They are as follows: Paranoia, by Dr. George Allen. Ancient and Modern Treatment of the Insane, by C. Spencer Kinney, M. D. General Paresis Mistaken for Chronic Alcoholism, by Daniel H. Arthur, M. D. And contribution to the General Pathology of the Insane, by Dr. Ales Hrdlicka.

